# Genomic Comparisons of Alphacoronaviruses and Betacoronaviruses from Korean Bats

**DOI:** 10.3390/v14071389

**Published:** 2022-06-25

**Authors:** Van Thi Lo, Sun Woo Yoon, Yong Gun Choi, Dae Gwin Jeong, Hye Kwon Kim

**Affiliations:** 1 Bionanotechnology Research Center, Korea Research Institute of Bioscience and Biotechnology, Daejeon 34141, Korea; van@kribb.re.kr (V.T.L.); syoon@kribb.re.kr (S.W.Y.); 2 Bio-Analytical Science Division, Korea University of Science and Technology (UST), Daejeon 34113, Korea; 3 The Korean Institute of Biospeleology, Daejeon 34225, Korea; kcavere@hanmail.net; 4 Department of Biological Sciences and Biotechnology, College of Natural Sciences, Chungbuk National University, Cheongju 28644, Korea

**Keywords:** bat-associated, Alphacoronavirus, MERS-related coronavirus, Korea

## Abstract

Coronaviruses are well known as a diverse family of viruses that affect a wide range of hosts. Since the outbreak of severe acute respiratory syndrome, a variety of bat-associated coronaviruses have been identified in many countries. However, they do not represent all the specific geographic locations of their hosts. In this study, full-length genomes representing newly identified bat coronaviruses in South Korea were obtained using an RNA sequencing approach. The analysis, based on genome structure, conserved replicase domains, spike gene, and nucleocapsid genes revealed that bat Alphacoronaviruses are from three different viral species. Among them, the newly identified B20-97 strain may represent a new putative species, closely related to PEDV. In addition, the newly-identified MERS-related coronavirus exhibited shared genomic nucleotide identities of less than 76.4% with other Merbecoviruses. Recombination analysis and multiple alignments of spike and RBD amino acid sequences suggested that this strain underwent recombination events and could possibly use hDPP4 molecules as its receptor. The bat SARS-related CoV B20-50 is unlikely to be able to use hACE2 as its receptor and lack of an open reading frame in ORF8 gene region. Our results illustrate the diversity of coronaviruses in Korean bats and their evolutionary relationships. The evolution of the bat coronaviruses related ORF8 accessory gene is also discussed.

## 1. Introduction

Bats have been recognized as a natural reservoir host and source of infection for several microorganisms, including several zoonotic viruses that can cause severe human diseases, such as Marburg, Nipah and Hendra viruses [1]. In addition, bats carry several other viruses with zoonotic potential that are genetically related to emerging human pathogens, including ebolaviruses and coronaviruses. Bats have a wide geographical distribution and the ability of flight [2]. The microbial transmission of coronaviruses from bats to wildlife and domestic animals and humans can occur both directly and indirectly. Some zoonotic viruses associated with bats have been identified, including pathogens that can cause severe human diseases, such as Ebola virus, Marburg virus, Nipah virus, and more recently, SARS-CoV-2 [1].

During the past two decades, numerous bat coronaviruses (BatCoVs) have been detected with a high prevalence and great genetic diversity worldwide [3]. They belong to the Alphacoronavirus (αCoV) and Betacoronavirus (βCoV) genera. Before the COVID-19 pandemic, a number of bat coronaviruses were identified with highly similar genomic sequences to human coronaviruses (HCoV-229E [4], HCoV-NL63 [5], SARS-CoV [6], and MERS-CoV [7]) and porcine coronaviruses (PEDV [8] and SADS-CoV [9]), which suggests that almost all human coronaviruses have zoonotic origins [10]. Afterward, bat coronaviruses that were closely related to SARS-CoV-2 were detected in bats from China and Southeast Asia [11,12,13]. However, predicting the potential pathogenicity of a newly discovered bat coronavirus is still difficult because most attempts to isolate bat coronaviruses have been unsuccessful [14].

Coronaviruses (CoVs) exhibit a large, positive, single-stranded RNA genome (~26–32 kb in length). They belong to the subfamily *Coronavirinae* under the family *Coronaviridae*. At present, CoVs are classified into four genera: Alphacoronavirus, Betacoronavirus, Gammacoronavirus, and Deltacoronavirus [15,16]. The complete genome contains five major open reading frames (ORFs) flanked by a 5′-untranslated region (UTR) and a 3′- UTR. The major ORFs encode replicase polyprotein (ORF1ab) and four structure proteins including spike glycoprotein (S), envelope protein (E), membrane protein (M), and nucleocapsid protein (N) [15]. A variable number of other ORFs that apparently encod nonstructural proteins were identified to be virus- or group-specific [17].

In South Korea, bat coronaviruses were identified based on their conserved partial RNA-dependent RNA polymerase (RdRp) gene sequences [18,19]. To date, two complete genomes of bat coronavirus have been described, including BatCoV HCQD-2020 and bat SARS-like CoV [20,21]. Hence, further studies are needed to understand the features of the complete genomic bat coronavirus representatives and their relationships. This will be essential to estimating their pathogen potential and evolution to prevent the spread of bat coronaviruses to humans.

In this study, we utilized a RNA-seq approach to complete genomic sequences of bat coronaviruses. The phylogenetic and genomic characteristics of these viruses were previously underestimated. Herein, we report newly described αCoVs, SARS-related, and newly-identified MERS-related coronaviruses. Furthermore, their complete genomic characteristics were analyzed to infer their evolutionary relationships with known viruses.

## 2. Materials and Methods

### 2.1. Sample Collection and RNA Isolation for RNA-Seq

Coronavirus-positive samples (Table 1) were detected from bat feces collected in South Korea from 2018 to 2021, as previously described [19]. The samples were confirmed positive in nested pan-CoV RT-PCR and sequencing [22].

The positive samples (10% (*w*/*v*) fecal samples in transport medium (Universal Transport Medium, Noble Biosciences™, Gyeonggi, Korea) were centrifuged at 14,000× *g* for 10 min at 4 °C and filtered with a 0.22 μm pore-size filter. The samples were treated with DNase I before RNA was extracted using a QIAamp viral RNA mini kit (Qiagen, Helden, Germany). Total isolated RNA was cleaned up and concentrated using the RNeasy Mini Kit (Qiagen). The total RNA was submitted to the Macrogen Co., Ltd. (Seoul, Korea) for cDNA library construction using a TruSeq stranded total RNA LT sample Prep Kit (Gold) following the manufacturer’s protocol. Sequencing was performed using a NovaSeq 6000 platform.

Bat species were identified using specific primers for the Cytochrome b gene as previously described [23].

### 2.2. De Novo Assembly of Genomic Sequences

The raw read data were imported into Geneious Prime version 2022.0.2 (https://www.geneious.com/, accessed on 10 January 2022) for data processing. Paired reads were created from R1 and R2 files. The reads were trimmed to remove adapter sequences and filtered to remove low-quality reads with the cutoff threshold for average base quality score, which was set at 30 using the BBDuk plugin version 38.84. The raw reads and trimmed reads were checked and rechecked with FASTQC.

The trimmed reads were assembled de novo in QIAGEN CLC Genomics Workbench (v21.0.5). The resulting contig sequences of greater than 20 kb were blasted and aligned to reference sequences retrieved from GenBank for coronaviruses and other viruses. The mapping reads were conducted in Geneious Prime. A consensus sequence was generated by mapping the trimmed reads to the full-genome-length contig coronaviruses. To map the reads, 5–10 iterations were performed with the high sensitivity settings, e.g., maximum mismatches per read, minimum overlap identity, maximum gap size. The consensus sequences were aligned with the de novo contig sequences to confirm the newly assembled complete genomes.

### 2.3. Virus Genome Annotation

The ORFs of assembled genomes were annotated using the closest related reference sequences retrieved from GenBank. For the newly identified virus genome, the potential ORFs were predicted and annotated by the conserved signature of coronaviruses and aligned with the closest matching sequences, which were identified using BLAST and Geneious Prime.

### 2.4. Phylogenetic Analysis

Coronavirus reference sequence sets representing the whole genome, ORFab1, S and N genes were downloaded from GenBank. Sequence alignments were performed using the MAFFT algorithm in Geneious Prime. The best-fit model of nt substitutions was determined using jModelTest2 [24]. Preliminary phylogenetic analyses were conducted using the Neighbor-Joining method based on the Tamura-Nei model. The main phylogenetic analyses were performed under a Bayesian statistical framework implemented in BEAST (version 1.10.4) [25], and were displayed in FigTree (version 1.4.4).

### 2.5. Recombination Analysis

Potential recombination events in the history of the identified bat coronaviruses were assessed using RDP4 [26] and SimPlot (v3.5.1) [27]. The RDP4 analysis was conducted based on the complete genome sequence, using RDP, GENECONV, BootScan, maximum chi square Chimera, SISCAN, and 3SEQ methods.

## 3. Results

### 3.1. Genome Organization and Sequence Similarity Analysis

#### 3.1.1. Newly Described Bat Alphacoronaviruses

The complete genomes of four αCoV strains of B20-97, B20-104-1, B20-104-2, and B20-177 were assembled and characterized.

**Figure 1:**
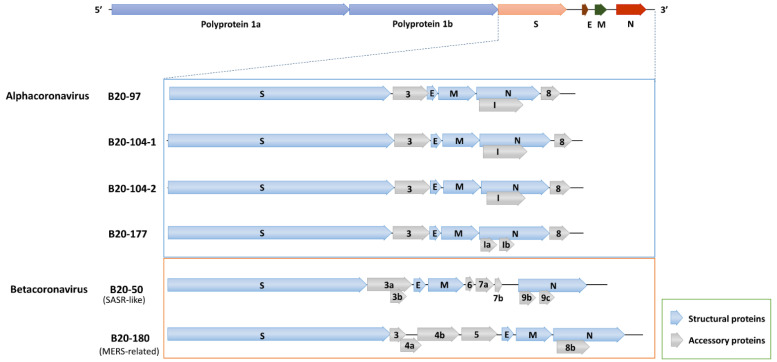
Genome organization of six strains. The general genomic structure is depicted at the top. The expanded regions below show the structural and accessory protein in the 3′ regions of the αCoVs (B20-97, B20-104-1, B20-104-2, and B20-177) and βCoVs (B20-50 and B20-180). The expanded regions are drawn to scale.

The lengths of the four αCoV genomes ranged between 27.8 kb and 28.1 kb with G + C contents between 40.8 and 43.8%. The assembled genome had classic coronavirus genome organization in which the replicase gene and structural protein genes were arranged in the order 5′ORF1ab-S-3-E-M-N-(8), as presented in Figure 1. The putative transcription regulatory sequence leader (TRS-L) motif was conserved for all identified genome sequences, 5′-CAACUAAACGAAAUU-3′. The TRS body (TRS-body) preceded each ORF, including ORF8, which is a putative ORF coding for unknown functional proteins (Table 2).

ORF3, located between the S and E genes, codes for an accessory protein. B20-97 ORF3 was predicted to encode a protein of 225 amino acids similar to the accessory membrane protein PEDV (predicted by Pfam, https://pfam.xfam.org, accessed on 30 March 2022). Both ORF3s of the B20-104-1 and B20-104-2 code a similar uncharacterized protein, which was identified by ORF3 of BtMr-SAX2011, with predicted proteins of 220 and 208 amino acids (aa), respectively. A non-structural protein 3 of B20-177, 226 aa long was identified as BatCoV-512 virus. Downstream of the N gene in all the identified αCoVs, there was an ORF named ORF8 that was predicted to encode the 112–131-aa protein.

#### 3.1.2. Sequence Similarity Analysis

**Figure 2:**
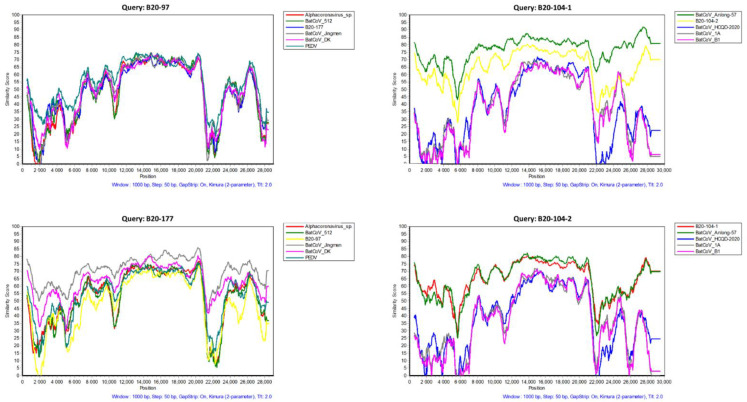
Genomic sequence identities between newly described bat CoVs and other closed αCoVs. The similarity identities were generated using Simplot (v3.5.1).

B20-97 isolated from *Myotis petax* bat was similar to the partial JTAC2 sequence (KU182966) from greater tube-nosed bats (*Murina leucogaster*) in China, with amino acid identities of 99.3% in the RdRp. However, the strain shared high identity values with PEDV with nucleotide identities of 67.4% in total genome and amino acid identities of 59.1% and 56.1% in the S and N sequences, respectively. The whole genome similarity is presented in Figure 2. A separate comparison of the amino acid sequences of seven conserved ORFab domains is presented in Table 3, as suggested by the International Committee on Taxonomy of Viruses (ICTV). A comparison of the amino acid sequences of the seven concatenated domains for coronavirus species demarcation showed that the BatCoV B20-97 possess <90% of amino acid identities compared to those of two closely related viruses (PEDV and BatCoV-512), indicating that B20-97 represents the new identified species of αCoV.

The complete sequences B20-104-1 and B20-104-2 were isolated from the total RNA extracted from one feces sample collected from *Rhinolophus ferrumequinum*, sharing 71.7% of their genomic nucleotide identities, and 63.1% and 71.2% of their amino acid identities in the S and N protein, respectively. They are both closely related to the strain Anlong-57 and BtMr-SAX2011, sharing 71–81% of their average nucleotide identities (Figure 2 and Table 3), respectively. The sequence identities of the concatenated domains of these two newly identified viruses and the *Myotis ricketti* Alphacoronavirus Sax-2011 are around 95% and 90% (B20-104-1 and B20-104-2, respectively), which suggests that they are the same viral species as proposed by the ICTV.

B20-177 was identified in *Myotis macrodactylus* belonging to the most abundant Alphacoronavirus K1 group in Korea, as in our previous study [19]. The complete genome shared the same features as almost all αCoVs. Notably, there are two open reading frames inside the N gene, which encode two possible internal proteins (Figure 1 and Table S1). The sequence was most closely related to the BatCoV-Jingmen isolate (Jingmen Miniopterus schreibersii alphacoronavirus 2, access number: MZ328300), which shared 72.4% of the complete genomic nucleotide identities and 75.4% and 69.1% amino acid similarity by aligning in the S and N residue amino acid sequences, respectively. With around 90% of the concatenated conserved domains of ORF1ab polyprotein, the B20-177 and BatCoV-Jingmen are supposed to be same viral species, but distinguished from PEDV species.

#### 3.1.3. SARS-Related and Newly Identified MERS-Related Coronaviruses

Two complete genome sequences of B20-50 and B20-180 were both obtained from feces of the same bat species (*Rhinolophus ferrumequinum*) collected in 2020.

**Figure 3:**
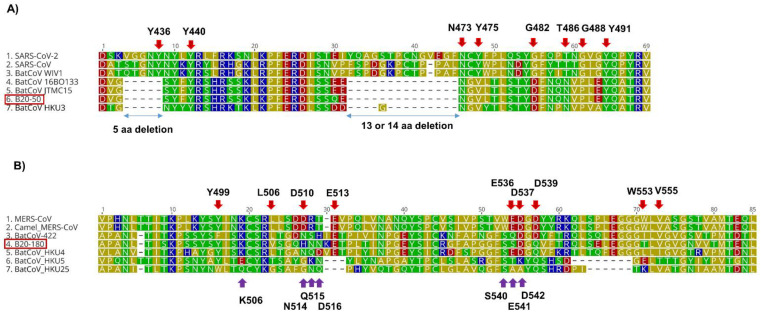
Multiple alignments of the amino acid sequence of the receptor-binding motif (RBM) of the spike protein of SARS-related CoVs (**A**) and Merbecoviruses (**B**). (**A**) Red arrows indicate the position with conserved residues binding between SARS-CoV and SARS-CoV-2 to human angiotensin-converting enzyme 2 (ACE2) in SARS-CoV based on [28]. (**B**) Red and purple arrows indicate the position with conserved residues binding to hDPP4 in MERS-CoV [29] and BatCoV HKU4 [7], respectively. Our sequence name was highlighted in the red box. Amino acid residues were colored by their polarity. Multiple alignments were performed by the MAFFT algorithm in Geneious Prime.

The SARS-related coronavirus B20-50 genome size was 29,612 nucleotides long and shared a similar genome layout with known SARS-related CoV viruses, including TRS-L and TRS-B sequences. The genome sequence of B20-50 shared a high nucleotide identity to the closely related BatCoV 16BO133 (KY938558) (97.6%), which was identified from a swab of *Rhinolophus ferrumequinum* bat in Korea [21]. They shared more than 99% and 99.6% amino acid similarities in the coronavirus conserved domains of the replicase polyprotein gene and S gene, respectively. B20-50 shared 74.6% and 70.4% of full spike amino acid identities to SARS-CoV (NC_004718) and SARS-CoV-2 (NC_045512), respectively. Two deletions of 5 and 14 amino acids were observed within the B20-50 receptor binding motif (RBM) (Figure 3).

Nine nucleotide deletions were observed in the gene ORF7b, resulting in an earlier stop codon. Thus, the ORF7b protein of B20-50 was only 46 amino acids long. No open reading frame was observed within 316nt following the ORF7b and preceding N gene, where the ORF8 or ORF8a/ORF8b gene of SARS-related CoVs is usually located, named *hypothetical ORF8*. Sequence multiple alignments of the region showed that both B20-50 and 16BO133 lacked an open reading frame and shared high nucleotide identities with SARS-CoV Tor2 (Figure S1 and Table S2).

The complete genomic sequence of B20-180 is 30110 nucleotides long, with a G + C content of 39.1%. The strain B20-180 exhibits a similar genomic structure to MERS-CoV (Table 3). The B20-180 sequence showed 76% nt identities to the closest sequence (MG021452: BtCoV/li/GD/2014-422) and ≤74% with MERS-CoV or the other Merbecoviruses. A separate comparison of the amino acid sequences of seven conserved ORF1ab domains and S and N proteins is presented in Table 3. According to the ICTV definition, the newly-identified B20-180 belongs to the MERS-related coronavirus species. The predicted RdRp sequences of B20-180 shared 94.4% amino acid identities with those of MERS-CoV. The full spike protein of B20-180 shared 68.2 to 77.0% aa identities with MERS-CoV, BatCoV HKU4, HKU5, HKU25, and 422 (Figure 3 and Table S4). The predicted receptor binding domain (RBD) of BatCoV B20-180 shared 69.9%, 60.8%, and 34.1% aa identities with BatCoV HKU4, MERS-CoV, and HKU5, respectively.

In the receptor binding motif (RBM) of BatCoV B20-180, five conserved residues for human dipeptidyl peptidase 4 (hDPP4) binding (Y499, L506, E513, D537 (D542 in HKU4), and V555 in MERS-RBD; K506, S540 in HKU4) were found (Figure 3).

### 3.2. Phylogenetic Analysis

Phylogenetic analyses based on the nucleotide sequences of the ORF1ab, S and N genes were conducted in BEAST (v1.10.4).

**Figure 4:**
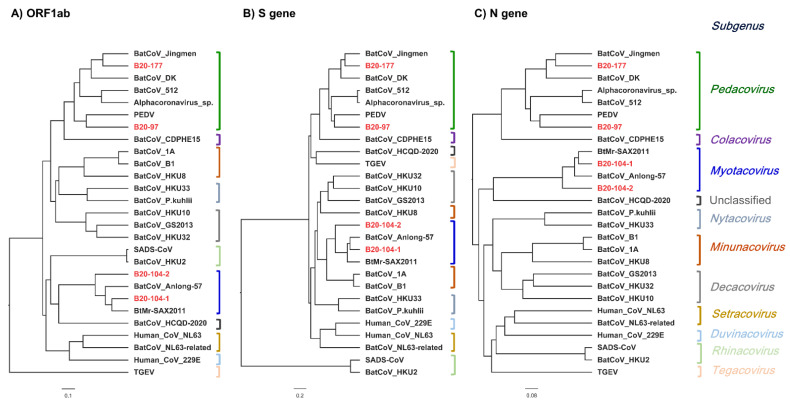
Maximum clade credibility (MCC) tree estimated by Bayesian Evolutionary Analysis Sampling Trees (BEAST) of full ORF gene from the complete genome sequences with reference sequence from αCoVs. Sequence names are provided in taxon labels. Strains identified in this study are colored in red. The (**A**–**C**) trees are based on ORF1ab, S, and N gene, respectively. The scales represent evolutionary distance. Statistical support (posterior probability) of all nodes was greater in value than 0.99.

In all the analyzed genes (Figure 4), B20-97 and B20-177 were most closely related to PEDV and BatCoV_Jingmen, respectively, and these viruses cluster with bat CDPHE15. Taken together, we suppose that B20-97 and B20-177 are putative species under subgenus Pedacovirus. In B20-104-1 and B20-104-2, they are closely related to BtMr-SAX2011 and BatCoV Anlong-57. In the ORF1ab gene and N gene trees, their species group clusters with a putative new species BatCoV HCQD-2020. However, they also cluster with BatCoV_1A in the S gene tree.

**Figure 5:**
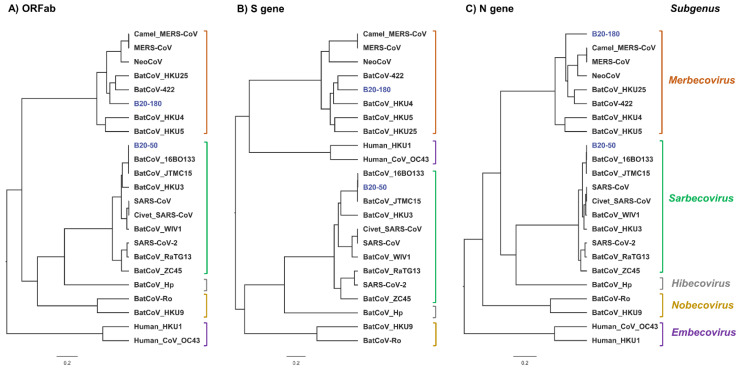
Maximum clade credibility (MCC) tree estimated with dated branches based on the full ORF gene from the complete genome sequences with reference sequence from βCoVs. Sequence names are provided in taxon labels. Strains identified in this study are colored in blue. The (**A**–**C**) trees are based on ORF1ab, S and N gene, respectively. Statistical support (posterior probability) of all nodes was greater in value than 0.98.

Phylogenetic trees (Figure 5) showed that B20-50 is closely related to SARS-related BatCoV-JTMC15 and BatCoV_16BO133, under the subgenus Sarbecovirus. The MERS-related B20-180 strain is more closely related to other bat MERS-related CoVs than MERS-CoV based on the ORF1ab gene tree. In contrast, the S gene tree showed that the strain is closely related to strain Bat-CoV-422 and, together, these cluster with HKU4 virus. Based on the N gene, the B20-180 is separated from all MERS-related CoVs within the Merbecovirus subgenus.

### 3.3. Recombination Analysis

Recombination detection was performed among genomes of each subgenus to which the identified strains belonged using the SimPlot software and RDP4. Possible recombination events were detected in the MERS-related B20-180 strain. To confirm the sequences, we designed five pairs of primers to obtain the S gene sequence of B20-180 using Sanger sequencing. The S gene sequences obtained by the Sanger sequencing matched the metagenomic sequences (Figure S2).

**Figure 6:**
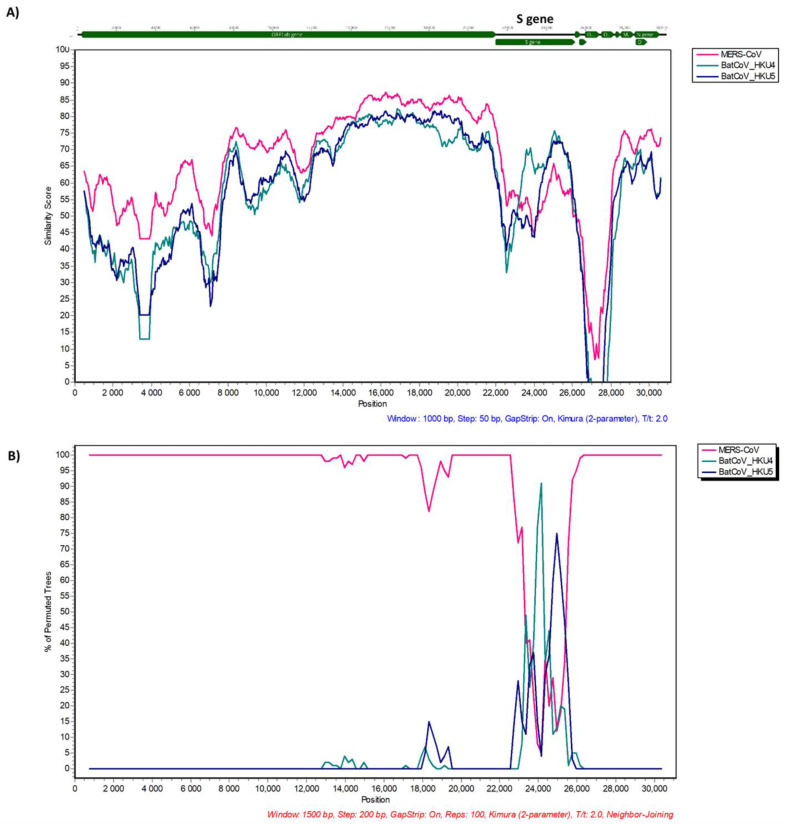
Comparison of genomic sequence identities between BatCoV B20-180 and other Merbecoviruses (**A**) and evidence for recombination events (**B**). The SimPlot and BootScan plots were generated using Simplot (v3.5.1).

We further focused on the spike protein, which is responsible for receptor binding and virus entry. Based on our analysis, recombination events were observed among the S gene of the B20-180 strain and two sequences belonging to the Merbecovirus subgenus. At least two breakpoints were identified among RBD and the S2 subunit of S gene by BootScan (RDP4), breakpoints at 24467–24742 (nt) from parent HKU4 and breakpoints at 24743–25154 (nt) from parent HKU5 as supported by BootScan *p*-value of 9.765 × 10^−10^ and 3.883 × 10^−10^, respectively. The potential recombination events were confirmed using the similarity plot and BootScan analyses implemented in Simplot (Figure 6).

## 4. Discussion

The emerging infectious diseases originating from zoonotic viruses significantly threaten global public health. Bats are one of the most important natural hosts of many zoonotic potential viruses that may cross the barriers to humans and livestock. The database covers more than 12,000 bat-associated viruses as of August 2021 [30]. Since the SARS-CoV epidemic, bats are the group of mammals in which the largest number of CoVs have been detected [3].

By the International Committee on Taxonomy of Viruses (2021), the Alphacoronavirus genus consists of 15 subgenera with 26 species, while Betacoronavirus includes five subgenera with 14 species. Most of these viral species have been found in bats, and it is suggested that all αCoVs originate from bats [31]. The genus αCoV is comprised of several viruses that cause infectious diseases in swine (including TGEV, PEDV, porcine respiratory coronavirus, and SADS-CoV) and humans (NL63-CoV and 229E-CoV). In this study, two identified αCoVs were defined as belonging to the same subgenus as PEDV. Recombinations and mutations occur at high frequencies in coronaviruses, especially recombination events within subgenera [32,33]. Therefore, the newly discovered viruses should be further studied to investigate their zoonotic potential. Synthetic recombinant viruses within subgenus could be used to investigate potential transmission mechanisms.

We successfully completed genomic descriptions of newly identified coronaviruses that were identified in different bat species, including *Myotis macrodactylus*, *Myotis petax*, and *Rhinolophus ferrumequinum*. In our previous study, several different bat species were observed to crow, co-roost or co-hibernate which increases the potential for intra- or interspecies transmission [19]. The B20-104-1 and B20-104-2 strains were co-infected in a sample collected from *Rhinolophus* bats, although they were identified as *Myotis ricketti* Alphacoronavirus Sax-2011, which supports the interspecies transmission of coronaviruses between bats.

In this study, four newly identified αCoVs are from three viral species belonging to two subgenera (Pedacovirus and Myotacovirus). BatCoV B20-104-1 and B20-104-2 are supposed to belong to the *Myotis ricketti* Alphacoronavirus Sax-2011. BatCoV B20-177 is supposed to be the same unclassified viral species under the Pedacovirus subgenus as BatCoV-Jingmen. BatCoV B20-97 represents a new putative species under the subgenus *Pedacovirus*. However, they have a similar genomic organization, containing ORF1ab-S-3-E-M-N-8. A putative ORF8 was found at the 3′ terminator of the genome, which encodes a heterologous protein of unknown function. The whole genome comparison and single gene analyses indicated that the B20-97 strain is more closely related to PEDV than BatCoV-512. However, the B20-97 strain has a similar genomic structure to BatCoV-512, as the PEDV genome lacks an ORF8 gene downstream of the N gene. Genome expansion is believed to be associated with virus adaptation to specific or new hosts in CoVs [34]. In addition, human coronaviruses could evolve through gene gains and losses [34]. We suggested that BatCoV B20-97 and PEDV shared the same origin and could be undertaking a different evolution pathway by adjusting the genome. This supports the hypothesis that PEDV may have originated from bats.

The emergence of SARS-CoV and SARS-CoV-2 has threatened global public health [35,36]. *Rhinolophus* bats are natural reservoirs of bat SARS-like coronaviruses [6]. The accessory gene of ORF8 is the second location variation in SARS-related coronavirus genomes after the S gene [10]. In the case of SARS-CoV, the ORF8 genes underwent adaptation during the transmission from animals to humans by losing genes [37]. Deletions in the ORF8 gene are associated with SARS-CoV-2 pathogenicity [38]. In the present study, the B20-50 genome lacks the open reading frame in the ORF8 gene region, similar to the bat SARS-related CoVs 16BO133 detected in Korea in 2016. A deletion (9 bp) downstream of the ORF7b gene of B20-50 led to the missing the start codon of the ORF8 gene. In contrast, the ORF8 gene of BatCoV JTMC15 (KU182964), found in the same bat species from China, was deleted. Moreover, large deletions were observed in the same region in SARS-CoV Tor2, B20-50, and BatCoV 16BO133 (highlighted in a red box, Figure S1), in which the deletion resulted in splitting the ORF8 of SARS-CoV in two ORF8a and ORF8b. The BatCoV B20-50 has one more deletion upstream of the *hypothetical ORF8* compared with BatCoV 16BO133. Thus, we hypothesize that the ORF8 may be evolving among bat SARS-related CoVs in Korea, with a tendency to undergo deletions.

Bat SARS-like CoV with two deletions in S sequences is unlikely to use ACE2 as a receptor for cell entry [39]. B20-50 spike RBD shared 3 of 8 conserved amino acid residues, in which the RBM of SARS-related CoVs interacts with the ACE2 receptor [28]. The BatCoV B20-50 is unlikely to be able to use ACE2 molecules as its receptor.

Some MERS-related coronaviruses belonging to the Merbecovirus subgenus can infect or circulate in humans, camels, and bats [40,41,42]. MERS-CoV is a highly pathogenic virus that causes an acute infectious disease of the respiratory system with a relatively high fatality ratio (approximately 35%) (https://www.who.int/, accessed on 15 March 2022). Bat coronavirus HKU4 and HKU25 related to MERS-CoV use the same human DPP4 receptor for cell entry [7,43,44]. We first described the complete genome of a bat MERS-related CoV from a fecal sample identified as *Rhinolophus ferrumequinum* bat in Korea. However, MERS-related CoVs are highly associated with *Vespertilionidae* bats [41]. We cannot exclude the possibility of cross-contamination because the sampling used environmental feces in multi-species sites. A further study on a sampling direct from a single bat is needed to confirm whether the *Rhinolophus* bat is a host of these strains or cross-contamination. MERS-CoV can undergo recombination events during frequent co-infection with distinct species [45]. We provided additional evidence for genetic recombination among the Merbecoviruses circulating in bat reservoirs. At least two recombination events were detected in the S gene of the newly identified MERS-related CoV B20-180. As a result, the S gene of BatCoV B20-180 is composed of three regions: the NTD region is more closely related to MERS-CoV; RBD and S2 subunits are acquired from HKU4 and HKU5, respectively. The RBM of B20-180 did not have two deletions (Figure 3). BatCoV HKU25 can use hDPP4 as its receptor for cell entry with lower efficiency than MERS-CoV and BatCoV HKU4 [43]. We found that the RBM of BatCoV B20-180 had more conserved residues binding to hDPP4 than that of BatCoV HKU25. The newly-identified MERS-related CoV could possibly use DPP4 as its receptor. More studies are essential to confirm and measure the receptor binding ability for human cell entry, such as protein-protein interactions, binding and pseudovirus assays.

## 5. Conclusions

The current study reveals the diversity features of the complete genome of bat-associated coronaviruses circulating in South Korea. The present study provides evidence for interspecies infection and the evolution pathway among Alphacoronaviruses. The genome data of newly described coronaviruses show a bigger picture of the diversity of coronaviruses in Korean bats. Furthermore, the study implies that newly identified MERS-related coronaviruses may have potential zoonosis, as they share the same host receptor as MERS-CoV due to recombination events. It is necessary to continue the long-term surveillance of these viruses and their genome analysis to predict zoonotic potential and prevent the future emergence of bat-borne infectious diseases. Thus, further studies should assess the zoonotic potential of these strains and the risk of cross-species transmission.

## Figures and Tables

**Table 1:** Bat sample information.

Sample No.	Identified Bat Species in Sample	Date of Collection	Location	Sample Type
B20-50	*Rhinolophus ferrumequinum*	30 March 2020	Jeonnam	Feces
B20-180	*Rhinolophus ferrumequinum*	20 September 2020	Gangwon	Feces
B20-97	*Myotis petax*	16 May 2020	Gangwon	Feces
B20-104	*Rhinolophus ferrumequinum*	18 May 2020	Gangwon	Feces
B20-177	*Myotis macrodactylus*	21 September 2020	Gangwon	Feces

**Table 2:** Putative transcription regulatory sequences (TRS) of six genome sequences (distance base to AUG).

Genome	TRS-L	TRS-B				
		S	E	M	N	Other ORFs
**Betacoronavirus**						
B20-50	AAACGAACUUUAAAAU (180)	AACGAA (1)	UACGAA (3)	AACGAA (45)	AACGAA (6)	ORF3: AACGAA (3) ORF7: AACGAA(1)
B20-180	UAACGAACUUAAAU (163)	AACGAA (46)	AACGAA (2)	AACGAA (10)	AACGAA (18)	ORF5: AACGAA (2) ORF4b: AACGAA (209) ORF3: AACGAU (7)
**Alphacoronavirus**						
B20-97	CAACUAAACGAAAUU (211)	AACUAU (8)	AACUAA (6)	AACGAA (0)	CUAAAC (6)	ORF3: AACUAA (10) ORF8: AACUAA (3)
B20-104-1	CAACUAAACGAAAUU (218)	AACUAA (5)	ACGAAA (23)	AACUAA (4)	AACUAA (3)	ORF3: AACUAA (39) ORF8: CGAAAU (61)
B20-104-1	CAACUAAACGAAAUU (217)	AACCAA (1)	AACUAACGAA (5)	AACUAA (4)	AACUAA (3)	ORF3: UACGAA (23) ORF8: UAAACG (0)
B20-177	CAACUAAACGAAAUU (213)	UACGAA (0)	GAAAUU (13)	AACGAA (0)	GAAAUU (0)	ORF3: AACUAA (42) ORF8: AACGAA (0)

**Table 3:** Comparison of newly-identified bat coronaviruses with related viruses in conserved replicase domains, structural proteins, and genomes (%).

Query Sequences	Close Sequences	% Nucleotide or Amino Acid Identity *										
		Genome	ADRP nsp3	3CLpro nsp5	RdRp nsp12	Hel nsp13	ExoN nsp14	NendoU nsp15	O-MT nsp16	S	N	Concatenate Domains
**Alphacoronavirus**												
B20-97	PEDV	67.4	70.4	78.7	90.0	93.7	89.2	84.9	92.3	59.1	56.1	87.3
	BatCoV_512	64.1	65.6	75.0	86.4	85.6	84.3	79.2	89.6	57.2	55.9	83.5
B20-104-1	BatCoV Anlong-57	80.5	83.3	91.9	96.9	97.9	94.1	91.0	97.0	83.2	84.5	94.6
	BtMr-SAX2011	81.2	85.6	92.9	97.5	96.8	95.5	91.6	96.3	82.5	82.6	95.3
B20-104-2	BatCoV Anlong-57	72.6	74.6	84.1	94.4	95.8	90.4	88.4	92.3	63.7	68.8	90.6
	BtMr-SAX2011	71.7	69.6	84.8	94.6	93.7	91.2	84.5	93.3	63.6	68.8	90.6
B20-177	BatCoV_Jingmen	72.4	76.0	85.5	91.1	95.8	93.1	92.8	97.4	75.4	69.1	90.8
	PEDV	67.2	72.0	77.0	86.7	88.5	85.7	81.9	91.9	60.1	55.1	85.4
**Betacoronavirus**												
B20-50	BatCoV 16BO133	97.6	99.2	99.3	99.9	100	100	100	100	99.6	99.3	99.8
B20-180	MERS-CoV	74.0	80.6	86.9	94.4	97.9	95.0	90.1	89.2	68.8	78.1	92.2
	HKU4	69.1	67.9	78.1	89.7	92.6	86.3	77.8	84.8	71.8	72.4	86.0

* Nucleotide identities are shown for genome. Amino acid identities are shown for other protein. PEDV: JQ023161; BatCoV Anlong-57: KY770851; BtMr-AlphaCoV/SAX2011: NC_028811; BatCoV_Jingmen: MZ328300; BatCoV 16BO133: KY938558; MERS-CoV: KF192507; HKU4: NC_009019.

## Data Availability

The genome sequences generated in this study were deposited into Genbank under accession numbers ON378802–ON378807. The RNA-seq data were deposited into the Sequence Read Archive (SRA) under accession number PRJNA833201.

## Supplementary Materials

The following supporting information can be downloaded at: https://www.mdpi.com/article/10.3390/v14071389/s1, Figure S1. Multiple alignment sequences of ORF7b and ORF8 gene regions of SARS-related CoVs; Table S1. Coding of potential of the coronavirus genome sequences; Table S2. Nucleotide identities (%) of ORF8 gene region of SARS-related CoVs; Table S3. Full Spike, RBD, and RBM amino acid identities (%) of BatCoV B20-50 to SARS-related CoVs; Table S4. Full Spike, RBD, and RBM amino acid identities (%) of BatCoV B20-180 to Merbecoviruses.

## References

[1] Allocati N., Petrucci A.G., Di Giovanni P., Masulli M., Di Ilio C., De Laurenzi V. (2016). Bat–man disease transmission: Zoonotic pathogens from wildlife reservoirs to human populations. Cell Death Discov..

[2] Wang L.-F., Anderson D.E. (2019). Viruses in bats and potential spillover to animals and humans. Curr. Opin. Virol..

[3] Wong A.C., Li X., Lau S.K., Woo P.C. (2019). Global epidemiology of bat coronaviruses. Viruses.

[4] Corman V.M., Baldwin H.J., Tateno A.F., Zerbinati R.M., Annan A., Owusu M., Nkrumah E.E., Maganga G.D., Oppong S., Adu-Sarkodie Y. (2015). Evidence for an ancestral association of human coronavirus 229E with bats. J. Virol..

[5] Huynh J., Li S., Yount B., Smith A., Sturges L., Olsen J.C., Nagel J., Johnson J.B., Agnihothram S., Gates J.E. (2012). Evidence supporting a zoonotic origin of human coronavirus strain NL63. J. Virol..

[6] Lau S.K., Woo P.C., Li K.S., Huang Y., Tsoi H.-W., Wong B.H., Wong S.S., Leung S.-Y., Chan K.-H., Yuen K.-Y. (2005). Severe acute respiratory syndrome coronavirus-like virus in Chinese horseshoe bats. Proc. Natl. Acad. Sci. USA.

[7] Wang Q., Qi J., Yuan Y., Xuan Y., Han P., Wan Y., Ji W., Li Y., Wu Y., Wang J. (2014). Bat origins of MERS-CoV supported by bat coronavirus HKU4 usage of human receptor CD26. Cell Host Microbe.

[8] Huang Y.-W., Dickerman A.W., Piñeyro P., Li L., Fang L., Kiehne R., Opriessnig T., Meng X.-J. (2013). Origin, evolution, and genotyping of emergent porcine epidemic diarrhea virus strains in the United States. mBio.

[9] Zhou P., Fan H., Lan T., Yang X.-L., Shi W.-F., Zhang W., Zhu Y., Zhang Y.-W., Xie Q.-M., Mani S. (2018). Fatal swine acute diarrhoea syndrome caused by an HKU2-related coronavirus of bat origin. Nature.

[10] Cui J., Li F., Shi Z.-L. (2019). Origin and evolution of pathogenic coronaviruses. Nat. Rev. Microbiol..

[11] Burki T. (2020). The origin of SARS-CoV-2. Lancet Infect. Dis..

[12] Delaune D., Hul V., Karlsson E.A., Hassanin A., Ou T.P., Baidaliuk A., Gámbaro F., Prot M., Tu V.T., Chea S. (2021). A novel SARS-CoV-2 related coronavirus in bats from Cambodia. Nat. Commun..

[13] Wacharapluesadee S., Tan C.W., Maneeorn P., Duengkae P., Zhu F., Joyjinda Y., Kaewpom T., Chia W.N., Ampoot W., Lim B.L. (2021). Evidence for SARS-CoV-2 related coronaviruses circulating in bats and pangolins in Southeast Asia. Nat. Commun..

[14] Banerjee A., Kulcsar K., Misra V., Frieman M., Mossman K. (2019). Bats and coronaviruses. Viruses.

[15] Brian D., Baric R. (2005). Coronavirus genome structure and replication. Coronavirus Replication and Reverse Genetics.

[16] De Groot R.J., Baker S.C., Baric R., Enjuanes L., Gorbalenya A., Holmes K.V., Perlman S., Rottier P.J., Talbot P.J., Woo P.C. (2011). Coronaviridae. Virus Taxonomy, Classification and Nomenclature of Viruses.

[17] Liu D.X., Fung T.S., Chong K.K.-L., Shukla A., Hilgenfeld R. (2014). Accessory proteins of SARS-CoV and other coronaviruses. Antivir. Res..

[18] Lee S., Jo S.-D., Son K., An I., Jeong J., Wang S.-J., Kim Y., Jheong W., Oem J.-K. (2018). Genetic characteristics of coronaviruses from Korean bats in 2016. Microb. Ecol..

[19] Lo V.T., Yoon S.W., Noh J.Y., Kim Y., Choi Y.G., Jeong D.G., Kim H.K. (2020). Long-term surveillance of bat coronaviruses in Korea: Diversity and distribution pattern. Transbound. Emerg. Dis..

[20] Do H.-Q., Nguyen V.-G., Chung C.-U., Jeon Y.-S., Shin S., Jang K.-C., Pham L.B.H., Kong A., Kim C.-U., Park Y.-H. (2021). Genomic Characterization of a Novel Alphacoronavirus Isolated from Bats, Korea, 2020. Viruses.

[21] Kim Y., Son K., Kim Y.-S., Lee S.-Y., Jheong W., Oem J.-K. (2019). Complete genome analysis of a SARS-like bat coronavirus identified in the Republic of Korea. Virus Genes.

[22] Chu D.K., Leung C.Y., Gilbert M., Joyner P.H., Ng E.M., Tse T.M., Guan Y., Peiris J.S., Poon L.L. (2011). Avian coronavirus in wild aquatic birds. J. Virol..

[23] Schlegel M., Ali H.S., Stieger N., Groschup M.H., Wolf R., Ulrich R.G. (2012). Molecular identification of small mammal species using novel cytochrome B gene-derived degenerated primers. Biochem. Genet..

[24] Darriba D., Taboada G.L., Doallo R., Posada D. (2012). jModelTest 2: More models, new heuristics and parallel computing. Nat. Methods.

[25] Drummond A.J., Rambaut A. (2007). BEAST: Bayesian evolutionary analysis by sampling trees. BMC Evol. Biol..

[26] Martin D.P., Murrell B., Golden M., Khoosal A., Muhire B. (2015). RDP4: Detection and analysis of recombination patterns in virus genomes. Virus Evol..

[27] Lole K.S., Bollinger R.C., Paranjape R.S., Gadkari D., Kulkarni S.S., Novak N.G., Ingersoll R., Sheppard H.W., Ray S.C. (1999). Full-length human immunodeficiency virus type 1 genomes from subtype C-infected seroconverters in India, with evidence of intersubtype recombination. J. Virol..

[28] Lan J., Ge J., Yu J., Shan S., Zhou H., Fan S., Zhang Q., Shi X., Wang Q., Zhang L. (2020). Structure of the SARS-CoV-2 spike receptor-binding domain bound to the ACE2 receptor. Nature.

[29] Wang N., Shi X., Jiang L., Zhang S., Wang D., Tong P., Guo D., Fu L., Cui Y., Liu X. (2013). Structure of MERS-CoV spike receptor-binding domain complexed with human receptor DPP4. Cell Res..

[30] Zhou S., Liu B., Han Y., Wang Y., Chen L., Wu Z., Yang J. (2022). ZOVER: The database of zoonotic and vector-borne viruses. Nucleic Acids Res..

[31] Woo P.C., Lau S.K., Lam C.S., Lau C.C., Tsang A.K., Lau J.H., Bai R., Teng J.L., Tsang C.C., Wang M. (2012). Discovery of seven novel Mammalian and avian coronaviruses in the genus deltacoronavirus supports bat coronaviruses as the gene source of alphacoronavirus and betacoronavirus and avian coronaviruses as the gene source of gammacoronavirus and deltacoronavirus. J. Virol..

[32] Makino S., Keck J.G., Stohlman S.A., Lai M. (1986). High-frequency RNA recombination of murine coronaviruses. J. Virol..

[33] Bobay L.-M., O’Donnell A.C., Ochman H. (2020). Recombination events are concentrated in the spike protein region of Betacoronaviruses. PLoS Genet..

[34] Forni D., Cagliani R., Clerici M., Sironi M. (2017). Molecular evolution of human coronavirus genomes. Trends Microbiol..

[35] Peiris J., Lai S., Poon L., Guan Y., Yam L., Lim W., Nicholls J., Yee W., Yan W., Cheung M. (2003). Coronavirus as a possible cause of severe acute respiratory syndrome. Lancet.

[36] Zhou P., Yang X.-L., Wang X.-G., Hu B., Zhang L., Zhang W., Si H.-R., Zhu Y., Li B., Huang C.-L. (2020). A pneumonia outbreak associated with a new coronavirus of probable bat origin. Nature.

[37] Zinzula L. (2021). Lost in deletion: The enigmatic ORF8 protein of SARS-CoV-2. Biochem. Biophys. Res. Commun..

[38] Su Y.C., Anderson D.E., Young B.E., Linster M., Zhu F., Jayakumar J., Zhuang Y., Kalimuddin S., Low J.G., Tan C.W. (2020). Discovery and genomic characterization of a 382-nucleotide deletion in ORF7b and ORF8 during the early evolution of SARS-CoV-2. mBio.

[39] Ren W., Qu X., Li W., Han Z., Yu M., Zhou P., Zhang S.-Y., Wang L.-F., Deng H., Shi Z. (2008). Difference in receptor usage between severe acute respiratory syndrome (SARS) coronavirus and SARS-like coronavirus of bat origin. J. Virol..

[40] Chu D.K., Poon L.L., Gomaa M.M., Shehata M.M., Perera R.A., Zeid D.A., El Rifay A.S., Siu L.Y., Guan Y., Webby R.J. (2014). MERS coronaviruses in dromedary camels, Egypt. Emerg. Infect. Dis..

[41] Luo C.-M., Wang N., Yang X.-L., Liu H.-Z., Zhang W., Li B., Hu B., Peng C., Geng Q.-B., Zhu G.-J. (2018). Discovery of novel bat coronaviruses in South China that use the same receptor as Middle East respiratory syndrome coronavirus. J. Virol..

[42] Zaki A.M., Van Boheemen S., Bestebroer T.M., Osterhaus A.D., Fouchier R.A. (2012). Isolation of a novel coronavirus from a man with pneumonia in Saudi Arabia. N. Engl. J. Med..

[43] Lau S.K., Zhang L., Luk H.K., Xiong L., Peng X., Li K.S., He X., Zhao P., Fan R.Y., Wong A.C. (2018). Receptor usage of a novel bat lineage C betacoronavirus reveals evolution of MERS-related coronavirus spike proteins for human DPP4 binding. J. Infect. Dis..

[44] Yang Y., Du L., Liu C., Wang L., Ma C., Tang J., Baric R.S., Jiang S., Li F. (2014). Receptor usage and cell entry of bat coronavirus HKU4 provide insight into bat-to-human transmission of MERS coronavirus. Proc. Natl. Acad. Sci. USA.

[45] Dudas G., Rambaut A. (2016). MERS-CoV recombination: Implications about the reservoir and potential for adaptation. Virus Evol..

