# Supplementary material for: Genomic Comparisons of Alphacoronaviruses and Betacoronaviruses from Korean Bats
Source: Viruses. 2022 Jun 25;14(7):1389. doi: 10.3390/v14071389 (PMC9320528; doi:10.3390/v14071389)
Supplement: Supplementary file 1 [file viruses-14-01389-s001.zip › viruses-1728928-supplementary.pdf]

Article

Genomic comparisons of Alphacoronaviruses and Betacoronaviruses from Korean bats

Van Thi Lo [1,4,†], Sun-woo Yoon [1,4,†] , Yong Gun Choi [3], Dae Gwin Jeong [1,4,*] and Hye Kwon Kim [2,*]

Supplementary Materials

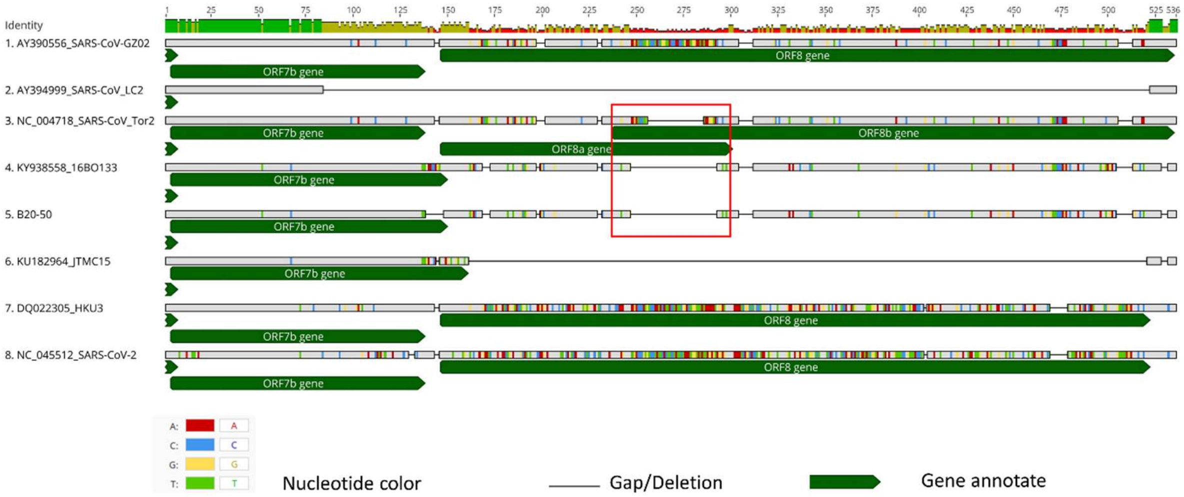

Figure S1. Multiple alignment sequences of ORF7b and ORF8 gene regions of SARS-related CoVs. All disagreenments of nucleotides were highlighted to consensus sequences were described by four colors representing for each nucleotide.

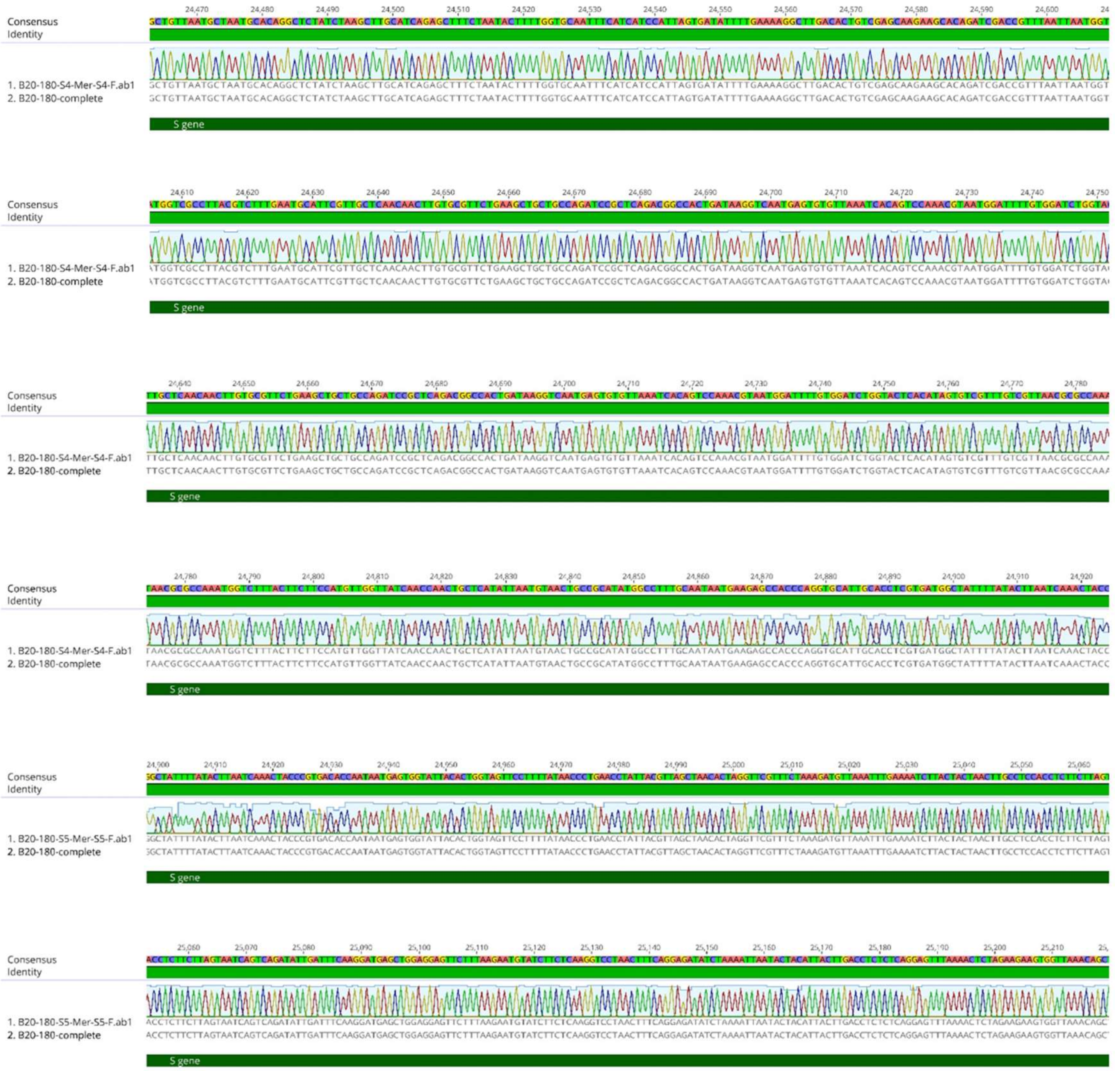

Figure S2. An alignment of Sanger sequencing sequences and metagenomic sequences of the BatCoV B20-50. Sequence position numbers were marked on the top of the consensus sequences.

Table S1. Coding of potential of the coronavirus genome sequences.

Gene	Start	End	Nucleotide length (bp)	Protein length (aa)
**Alphacoronavirus**				
**B20-97: 28,162 nt – 43.8% GC**				
**ORF1ab gene**	278	20,646	20,369	6790
**S gene**	20,646	24,743	4,098	1366
**ORF3 gene**	24,766	25,440	675	225
**E gene**	25,421	25,651	231	77
**M gene**	25,657	26,337	681	227
**N gene**	26,346	27,536	1,191	397
**N gene:** possible internal protein (I)	26,401	27,210	810	270
**ORF8 gene**	27,549	27,938	390	130
**B20-104-1: 28,159 nt – 42.2% GC**				
**ORF1ab gene**	286	20,495	20,210	6737
**S gene**	20,497	24,669	4,173	1391
**ORF3 gene**	24,666	25,325	660	220
**E gene**	25,332	25,562	231	77
**M gene**	25,572	26,258	687	229
**N gene**	26,267	27,559	1,293	431
**N gene:** possible internal protein (I)	26,379	27,062	684	228
**ORF8 gene**	27,633	27,968	336	112
**B20-104-2: 27836 nt – 43.4% GC**				
**ORF1ab gene**	285	20,215	19,931	6644
**S gene**	20,221	24,387	4,167	1389
**ORF3 gene**	24,384	25,009	626	208
**E gene**	25,016	25,246	231	77
**M gene**	25,256	25,939	684	228
**N gene**	25,948	27,207	1,260	420
**N gene:** possible internal protein (I)	26,060	26,731	672	224
**ORF8 gene**	27,211	27,603	393	131
**B20-177: 28.187 nt – 40.8% GC**				

**ORF1ab gene**	281	20,604	20,324	6775
**S gene**	20,601	24,692	4,092	1364
**ORF3 gene**	24,692	25,369	678	226
**E gene**	25,350	25,580	231	77
**M gene**	25,586	26,269	684	228
**N gene**	26,279	27,556	1,278	426
**N gene: possible internal protein (Ia)**	26,337	26,618	282	94
**N gene: hypothesis protein (Ib)**	26,679	26,915	237	79
**ORF8 gene**	27,562	27,933	372	124
**Betacoronavirus**				
**B20-50 (SARS-like): 29,612 nt – 40.9% GC**				
**ORF1ab gene**	247	21,452	21,207	7069
**S gene**	21,459	25,169	3,711	1237
**ORF3a gene**	25,178	26,002	825	275
**ORF3b gene**	25,599	25,943	345	115
**E gene**	26,027	26,257	231	77
**M gene**	26,308	26,973	666	222
**ORF6 gene**	26,984	27,174	191	63
**ORF7a gene**	27,182	27,550	369	123
**ORF7b gene**	27,547	27,684	138	46
**N gene**	28,001	29,263	1,263	421
**ORF9b gene**	28,011	28,304	294	89
**ORF9c gene**	28,461	28,673	213	71
**B20-180 (MERS-related): 30,110 nt – 39.1% GC**				
**ORF1ab gene**	226	21,446	21,221	7074
**S gene**	21,388	25,455	4,068	1356
**ORF3 gene**	25,468	25,758	291	97
**ORF4a gene**	25,688	26,050	363	121
**ORF4b gene**	25,977	26,732	756	252
**ORF5 gene**	26,805	27,467	663	221
**E gene**	27,547	27,795	249	83
**M gene**	27,810	28,472	663	221

**N gene**	28,524	29,810	1,287	429
**ORF8b gene**	28,570	29,145	576	192

Table S2. Nucleotide identities (%) of ORF8 gene region of SARS-related CoVs.

Sequences	**B20-50**	**B16BO133**	**SARS-CoV-Tor2**	**SARS-CoV-GZ02**	**HKU3**
B20-50					
B16BO133	98.7				
SARS-CoV-Tor2	74.0	73.2			
SARS-CoV-GZ02	68.7	68.0	91.6		
HKU3	35.5	35.7	41.4	45.8	
SARS-CoV-2-WH1	35.5	35.7	38.2	43.2	66.4

Table S3. Full Spike, RBD, and RBM amino acid identities (%) of BatCoV B20-50 to SARS-related CoVs.

**Sequences**	**B16BO133**	**BatCoV JTMC15**	**BatCoV HKU3**	**BatCoV WIV1**	**SARS-CoV**	**SARS-CoV-2**
Full Spike	99.6	99.6	80.1	74.8	74.6	70.4
RBD	99.4	99.4	88.7	61.9	61.3	62.1
RBM	98.0	98.0	76.5	32.4	30.9	31.9

Table S4. Full Spike, RBD, and RBM amino acid identities (%) of BatCoV B20-180 to Merbecoviruses.

**Sequences**	**MERS-CoV**	**Camel MERS-CoV**	**BatCoV-422**	**HKU4**	**HKU5**	**HKU25**
Full Spike	68.8	68.2	77.0	71.8	70.0	70.0
RBD	60.8	60.4	81.1	69.9	57.6	59.8
RBM	47.1	47.1	71.4	65.5	34.1	35.7
